# Electrophysiological Evidence of Contralateral Neuromuscular Effects Following Long-Term Botulinum Toxin Therapy in Hemifacial Spasm

**DOI:** 10.3390/toxins17080407

**Published:** 2025-08-14

**Authors:** Tehran Aliyeva, Mehmet Fevzi Oztekin, Yasemin Eren, Zeynep Nese Oztekin

**Affiliations:** 1 Department of Neurology, Faculty of Medicine, Istanbul Medipol University, 34000 Istanbul, Turkey; 2 Department of Neurology, Ankara Etlik City Hospital, University of Health Sciences, 06000 Ankara, Turkey; 3 Department of Neurology, Ankara City Hospital, University of Health Sciences, 06000 Ankara, Turkey

**Keywords:** hemifacial spasm, botulinum toxin, neuromuscular transmission, single-fiber electromyography, CMAP, diffusion, concentric needle electrode, electroneuromyography

## Abstract

Hemifacial spasm (HFS) is a cranial nerve disorder characterized by involuntary contractions of muscles innervated by the facial nerve. Botulinum toxin type A (BoNT-A) is widely used for symptom control. Although local diffusion is well established, the extent and clinical relevance of BoNT-A spread to contralateral muscles remain unclear. This study aimed to investigate the contralateral neuromuscular effects of BoNT-A in patients undergoing long-term treatment with BoNT-A. This retrospective cross-sectional study included 39 patients with HFS (mean age, 58.6 ± 8.5 years). Bilateral compound muscle action potentials (CMAPs) were recorded before and four weeks after the BoNT-A injection. Single-fiber electromyography (SFEMG) jitter and mean consecutive difference (MCD) were evaluated contralaterally using concentric needle electrodes. Patients were categorized as first-time (*n* = 10) or long-term (*n* = 29; treatment duration: 1–20 years) BoNT-A recipients. Contralateral CMAP amplitudes decreased by 21.1% post-injection (*p* < 0.001). MCD increased from 33.2 ± 5.6 to 37.0 ± 5.3 µs (*p* < 0.001), and jitter rose by 81%, from 7.9 ± 6.2 to 14.3 ± 8.1 µs (*p* < 0.001). The percentage increase in MCD was significantly higher in long-term versus first-time patients (12.7% vs. 7.5%; *p* = 0.039), suggesting a cumulative neuromuscular effect. Spontaneous myokymia or fasciculations were clinically observed in four long-term patients. These findings provide electrophysiological evidence that unilateral BoNT-A injections may induce neuromuscular transmission abnormalities in the contralateral facial muscles. This effect appears more pronounced in chronically treated individuals, highlighting the need for awareness of potential bilateral spread when planning long-term therapy.

## 1. Introduction

Hemifacial spasm (HFS) is a peripheral craniofacial movement disorder characterized by unilateral, intermittent, and involuntary contractions of muscles innervated by the ipsilateral facial nerve [1,2,3]. Symptoms typically begin in the orbicularis oculi and may extend to involve additional muscles such as the zygomaticus, buccinator, and platysma [1,2]. Although benign in its clinical course, HFS can substantially impair health-related quality of life due to visual disturbances, speech interference, and social embarrassment [1,3].

Epidemiological estimates suggest that HFS affects approximately 11 individuals per 100,000, with symptom onset most commonly occurring in the fifth or sixth decade of life [2,3]. The most frequently implicated pathophysiological mechanism is neurovascular compression at the root exit zone (REZ) of the facial nerve, typically caused by the anterior inferior cerebellar artery (AICA), posterior inferior cerebellar artery (PICA), or vertebral artery [4,5]. This compression can result in focal demyelination, ephaptic transmission, and increased neuronal excitability. Less frequently, structural lesions such as meningiomas or vestibular schwannomas may underlie the disorder [6,7].

Two principal treatment modalities are currently available: microvascular decompression (MVD) and botulinum toxin type A (BoNT-A) injections. MVD remains the only potentially curative intervention, demonstrating long-term success rates exceeding 90% in large surgical series [6]. However, its invasiveness, perioperative risks, and the requirement for specialized microsurgical expertise limit its applicability in broader clinical settings. BoNT-A injection, by contrast, provides temporary symptomatic relief and is considered a palliative treatment, particularly suitable for patients who are elderly or not candidates for surgery [8,9]. The therapeutic effect is mediated through inhibition of acetylcholine release at the neuromuscular junction, resulting in reversible chemodenervation that typically lasts 3–5 months [10,11,12].

Although BoNT-A is formulated for localized action, adverse effects such as ptosis, dysphagia, and unintended weakness in non-injected muscles have raised concerns regarding toxin diffusion, systemic migration, and retrograde axonal transport [13,14,15]. To date, most published data have relied on clinical observation, with few studies employing sensitive neurophysiological techniques to assess distant or contralateral effects objectively. Furthermore, many of these investigations are limited by small sample sizes, insufficient follow-up durations, or inadequate electrophysiological resolution, thereby leaving this clinically relevant phenomenon underexplored [16,17,18].

This gap in evidence is particularly salient given Toxins’ ongoing focus on the pharmacokinetics, dose–response relationships, and safety profiles of therapeutic neurotoxins. Clarifying whether repeated unilateral BoNT-A injections lead to contralateral neuromuscular transmission abnormalities is critical for optimizing injection protocols and mitigating potential risks, especially in patients undergoing chronic therapy.

Although the local diffusion of BoNT-A has been demonstrated in both animal models and clinical studies, the extent, temporal profile, and spatial distribution of its spread, particularly following repeated dosing, remain poorly characterized [11]. Electrophysiological techniques such as concentric needle single-fiber electromyography (SFEMG) and compound muscle action potential (CMAP) measurements provide high-resolution, objective markers of neuromuscular transmission integrity. These tools may aid in distinguishing actual diffusion-related effects from underlying bilateral excitability [14,16,18].

To address this critical gap, the present study investigates whether long-term, unilateral BoNT-A therapy induces subclinical neuromuscular changes in the contralateral orbicularis oculi muscle. Using CMAP amplitude and SFEMG jitter variability as outcome parameters, this work aims to contribute to the safety characterization of BoNT-A and guide future strategies for dose titration, injection intervals, and diffusion monitoring in facial movement disorders.

## 2. Materials and Methods

### 2.1. Study Design and Participants

This retrospective cross-sectional study included 39 patients (19 females and 20 males; age range: 18–65 years) diagnosed with hemifacial spasm (HFS). All participants were consecutively recruited from the Neurology Department of Diskapi Yildirim Beyazit Training and Research Hospital between 1 September 2015, and 6 June 2016; 06000 Ankara, Turkey

Inclusion criteria were as follows:

- A confirmed diagnosis of HFS based on clinical neurological examination.
- A minimum of three months since the most recent botulinum toxin type A (BoNT-A) injection, to eliminate acute-phase interference with neuromuscular assessments.
- Exclusion criteria included:
- Known neuromuscular disorders (e.g., myasthenia gravis).
- History of facial trauma, cosmetic facial surgery, or permanent facial implants.
- Active systemic or localized infections.
- Prior history of peripheral facial palsy (due to potential neuromuscular remodeling unrelated to BoNT-A effects).
- Coagulopathies or current use of anticoagulant or muscle relaxant medications.
- Pregnancy or lactation, due to potential hormonal effects on neuromuscular physiology and associated ethical considerations.

### 2.2. Ethical Approval

All participants provided written informed consent prior to enrollment. The study protocol was approved by the Institutional Ethics Committee of Diskapi Yildirim Beyazit Training and Research Hospital (Approval No: 31/23; dated 27 June 2016), and the study was conducted in accordance with the Declaration of Helsinki.

### 2.3. BoNT-A Injection Protocol

Onabotulinumtoxin A (Botox^®^, Allergan Inc., Irvine, CA, USA) was reconstituted in 0.9% preservative-free saline. A standardized dose of 25 units was administered at five injection points targeting the orbicularis oculi muscle on the affected side.

### 2.4. Electrophysiological Assessments

Electrophysiological studies were conducted using a Keypoint™ portable EMG system (Medtronic, Skovlunde, Denmark).

### 2.5. CMAP Recordings

Compound muscle action potentials (CMAPs) were bilaterally recorded from the orbicularis oculi muscles. Stimulation was applied supramaximally at the tragus level, targeting the zygomatic branch of the facial nerve. Surface recording electrodes were placed over the belly of the orbicularis oculi to capture responses.

### 2.6. Single-Fiber Electromyography (SFEMG) and Jitter Analysis

SFEMG was performed using disposable concentric needle electrodes (30 G, 25 × 0.33 mm; Alpine Biomed), which were integrated with the Keypoint™ system’s dedicated software module. Bandpass filter settings were adjusted to 1000 Hz–10 kHz to optimize signal fidelity.

Recordings were obtained from the contralateral orbicularis oculi muscle in all patients before and 4 weeks after the BoNT-A injection. For each subject, 20 fiber pairs were analyzed.

Jitter values and mean consecutive difference (MCD) were calculated automatically. The following criteria were used to define abnormal jitter:

- An MCD value greater than 34 µs was considered pathological.
- In addition, if ≥2 out of 20 fiber pairs exhibited jitter >44 µs, the result was also regarded as abnormal.

These thresholds are consistent with published normative data for concentric needle SFEMG in facial muscles [18,19]. While MCD reflects the average jitter, the inclusion of per-fiber pair cutoffs enhances the sensitivity of the analysis.

The analysis focused on group-level mean MCD values to assess whether long-term unilateral BoNT-A treatment may lead to cumulative, subclinical neuromuscular involvement in the contralateral side. Additionally, spontaneous activity such as fasciculations or myokymia was clinically monitored and documented when observed during the procedure.

## 3. Results

A total of 39 patients diagnosed with hemifacial spasm (HFS) were included in the study, comprising 19 females (48.7%) and 20 males (51.3%). The mean age of the participants was 58.6 ± 8.5 years (range, 29–65 years). Patients were divided into two subgroups based on treatment history:

- First-time BoNT-A recipients: *n* = 10 (25.6%)
- Recurrent BoNT-A recipients: *n* = 29 (74.4%)

The mean duration of BoNT-A therapy in the recurrent group was 6.2 ± 5.5 years (range, 1–20 years) (Table 1).

### 3.1. CMAP Amplitude Changes

Significant reductions in compound muscle action potential (CMAP) amplitudes were observed bilaterally following BoNT-A injection:

- Injected (affected) side: mean amplitude decreased from 1.5 ± 0.6 mV to 1.0 ± 0.4 mV, representing a 33.3% reduction (*p* < 0.001).
- Contralateral (non-injected) side: amplitude declined from 1.9 ± 0.7 mV to 1.5 ± 0.5 mV, indicating a 21.1% reduction (*p* < 0.001).

Although the reduction was more pronounced on the injected side (12.2% greater), the inter-lateral difference was not statistically significant (*p* = 0.501, Table 2).

CMAPs were recorded bilaterally from the orbicularis oculi muscle. The facial nerve was stimulated supramaximally at the anterior tragus using a manual stimulator; surface recording and reference electrodes were placed on the orbicularis oculi muscle.

### 3.2. MCD and Jitter Changes (Contralateral Side)

Following BoNT-A injection, both mean consecutive difference (MCD) and jitter values recorded from the contralateral orbicularis oculi significantly increased (Table 3).

- MCD: increased by 26.2%, from 28.6 ± 5.2 µs to 36.1 ± 6.8 µs (*p* < 0.001).
- Jitter: increased by 27.9%, from 30.1 ± 4.9 µs to 38.5 ± 7.2 µs (*p* < 0.001).

At baseline, 10.2% of patients (*n* = 4) had MCD values exceeding the abnormality threshold (greater than 34 µs). Post-injection, this proportion rose to 53.8% (*n* = 21), reflecting a more than fivefold increase in the prevalence of abnormal values.

### 3.3. Subgroup Analysis: First-Time vs. Recurrent BoNT-A Recipients

#### 3.3.1. Contralateral MCD:

- Pre-injection: first-time recipients: 26.2 ± 3.8 µs; recurrent recipients: 29.5 ± 5.1 µs (*p* = 0.009)
- Post-injection: first-time: 32.4 ± 4.6 µs; recurrent: 37.6 ± 6.9 µs (*p* < 0.001)
- Within-group increases:
- First-time: +23.7% (*p* = 0.049)
- Recurrent: +27.5% (*p* < 0.001)
- Between-group difference in increase: *p* = 0.039

#### 3.3.2. Contralateral Jitter:

- Pre-injection: first-time: 27.8 ± 3.5 µs; recurrent: 31.2 ± 5.1 µs (*p* < 0.001)
- Post-injection: first-time: 33.2 ± 4.9 µs; recurrent: 39.5 ± 7.0 µs (*p* < 0.001)
- Within-group increases:
  ○ First-time: +19.4% (*p* = 0.014)
  ○ Recurrent: +26.6% (*p* < 0.001)
- Between-group difference in increase: *p* = 0.009 (Table 4)

## 4. Discussion

This study provides objective electrophysiological evidence that unilateral botulinum toxin type A (BoNT-A) injections can induce subclinical neuromuscular transmission abnormalities in contralateral facial muscles, specifically the orbicularis oculi, in patients with hemifacial spasm (HFS). Notably, patients undergoing long-term treatment (1–20 years) exhibited significantly greater increases in single-fiber EMG jitter and mean consecutive difference (MCD) values, along with subtle reductions in contralateral compound muscle action potential (CMAP) amplitudes. These findings suggest a cumulative or systemic effect of BoNT-A, despite its intended local mechanism of action.

Emerging studies have reported that BoNT-A may spread beyond the injection site through passive diffusion, hematogenous dissemination, or retrograde axonal transport [13,14,20,21]. Although contralateral involvement is considered uncommon, several case reports and observational studies have documented neuromuscular effects in non-injected regions following facial or cervical injections [22,23,24,25,26]. However, most of these reports relied on clinical observation or low-resolution methods. In contrast, our study utilizes high-resolution electrophysiological parameters (CMAP and SFEMG) under standardized conditions, providing more robust evidence of subtle contralateral effects.

The observed decrease in contralateral CMAP amplitude is particularly noteworthy. While a reduction in CMAP amplitude on the injected side is a well-established effect of BoNT-A administration [12], similar changes on the non-injected side are uncommon. This observation may indicate subclinical diffusion of toxins or adaptive reorganization within bilateral facial motor circuits. Our finding is consistent with prior reports that have documented neuromuscular transmission alterations in muscles distant from the injection site, suggesting a more widespread or network-based response to repeated toxin exposure [15,20,23,27].

The marked increase in jitter and MCD values observed on the contralateral side provides further evidence of subclinical neuromuscular transmission disturbances. Single-fiber EMG is a sensitive method capable of detecting early synaptic abnormalities before clinical manifestations emerge [28]. The more pronounced jitter elevations in patients receiving long-term BoNT-A therapy support the hypothesis of cumulative dose effects and ongoing synaptic remodeling. Punga et al. [23] similarly demonstrated persistent jitter abnormalities in facial muscles not directly targeted by injections, even months after treatment. Our findings also align with emerging critical viewpoints emphasizing that the neuromuscular impact of BoNT-A on facial muscles—particularly outside the injection site—may be underrecognized in clinical practice [26,27].

Repeated cycles of chemodenervation and subsequent reinnervation may induce chronic alterations in motor endplate morphology, acetylcholine receptor density, and overall neuromuscular junction stability [10,11]. In our cohort, spontaneous muscle activity—including myokymia and fasciculations—was observed in a subset of patients undergoing long-term BoNT-A treatment. Although clinically subtle, these findings may reflect underlying synaptic reorganization, axonal sprouting, or heightened cholinergic receptor sensitivity [10], which have been proposed as mechanisms contributing to BoNT-A–induced plasticity.

Anatomically, upper facial muscles—such as the orbicularis oculi—receive partial bilateral corticobulbar innervation. This dual input may facilitate transhemispheric compensatory mechanisms or unmask previously silent motor pathways, especially in the context of repeated unilateral chemodenervation. Such neuroplastic responses have been proposed as potential contributors to contralateral facial hyperexcitability in long-term BoNT-A recipients [29].

### 4.1. Clinical Implications

From a clinical standpoint, these findings underscore the importance of recognizing potential bilateral effects when planning long-term botulinum toxin type A (BoNT-A) therapy. Although the observed electrophysiological changes were subclinical, they may have practical implications for dose titration, injection interval optimization, and muscle targeting, particularly in patients with limited neuromuscular reserve or bilateral predisposition. Incorporating routine electrophysiological monitoring in chronic BoNT-A users may enhance early detection of cumulative effects and contribute to more personalized and safer treatment strategies.

### 4.2. Limitations

This study has several limitations that should be acknowledged. First, the overall sample size, particularly within the subgroup of first-time BoNT-A recipients, was relatively modest, which may limit the generalizability of subgroup analyses. Second, electrophysiological assessments were confined to the orbicularis oculi muscle, which may not capture broader patterns of neuromuscular transmission alterations in other facial regions. Future studies incorporating additional muscle groups could provide a more comprehensive understanding of potential diffusion or systemic effects. Third, while concentric needle single-fiber electromyography (SFEMG) has been validated as a reliable technique [18], its resolution is inherently lower compared to classical single-fiber electrode methods, which may influence the sensitivity of jitter detection.

### 4.3. Future Directions

To advance understanding of contralateral neuromuscular effects and optimize long-term BoNT-A therapy, future research should address the following:

1. Longitudinal cohort studies tracking neuromuscular transmission parameters across multiple treatment cycles to evaluate cumulative effects.
2. Combined functional imaging and neurophysiological approaches, such as transcranial magnetic stimulation (TMS) and functional magnetic resonance imaging (fMRI), should be used to characterize bilateral facial motor circuit involvement.
3. Correlative histological investigations exploring patterns of denervation and reinnervation at the neuromuscular junction following repeated toxin exposure.
4. Comparative dosing strategies, including booster versus maintenance protocols, to determine optimal regimens that reduce unintended diffusion while maintaining efficacy.

## 5. Conclusions

This study is distinguished by its standardized BoNT-A dosing protocol, inclusion of both first-time and long-term recipients, and the use of objective electrophysiological measurements to assess contralateral neuromuscular effects. The combined observation of subclinical transmission abnormalities and spontaneous muscle activity on the non-injected side provides compelling evidence of regional toxin dissemination. Our findings demonstrate that unilateral BoNT-A administration may induce measurable neuromuscular changes in the contralateral facial muscles, particularly with prolonged exposure. These results underscore the importance of individualized dose titration, careful injection planning, and long-term electrophysiological monitoring in patients undergoing chronic BoNT-A therapy. As the clinical and cosmetic indications for botulinum toxin continue to expand, future prospective and mechanistic studies are essential to better characterize its diffusion dynamics and optimize patient safety.

## Figures and Tables

**Table 1:** Demographic and treatment characteristics of patients with hemifacial spasm who received unilateral BoNT-A injections.

Variable	Min–Max/*n* (%)	Median	Mean ± SD
Age (years)	29–65	63	58.6 ± 8.5
Treatment duration (years)	1–20	5	6.2 ± 5.5
Gender: Male	19 (48.7%)		
Gender: Female	20 (51.3%)		

Values are presented as mean ± standard deviation or *n* (%) unless otherwise indicated.

**Table 2:** Comparison of CMAP amplitudes in the orbicularis oculi muscles before and after BoNT-A injection on the injected (affected) and contralateral sides.

Amplitude	Affected Side			Contralateral Side			*p*
	Mean ± SD	Med (Min–Max)		Mean ± SD	Med (Min–Max)		
Before BoNT-A	1.5 ± 0.6	1.5	0.6–3.4	1.9 ± 0.7	1.8	0.9–3.7	0.000 ^w^
After BoNT-A	1.0 ± 0.4	1.0	0.4–2.5	1.5 ± 0.5	1.4	0.6–2.9	0.000 ^w^
Before/After BoNT-A	0.5 ± 0.4	0.4	0.0–1.8	0.5 ± 0.6	0.4	0.7–2.5	0.501 ^w^
Before/After BoNT-A *p*	0.000 ^w^			0.000 ^w^			

Data are presented as mean ± SD and median (min–max). ^w^: Wilcoxon signed-rank test.

**Table 3:** Electrophysiological changes in mean consecutive difference (MCD) and jitter values of the contralateral orbicularis oculi muscle before and after BoNT-A injection.

Measurement Parameter	Mean ± SD	Median	Min–Max	*p*
Contralateral Side MCD				
Before BoNT-A	33.2 ± 5.6	33	22–44	0.000 ^w^
After BoNT-A	37.0 ± 5.3	36	28–48	
Contralateral Side Jitter				
Before BoNT-A	7.9 ± 6.2	5	0–24	0.000 ^w^
After BoNT-A	14.3 ± 8.1	14	0–30	

^w^: Wilcoxon test; MCD: mean consecutive difference; BoNT: botulinum toxin; Measurements were obtained using concentric needle EMG. Values are mean ± SD and median (min–max). An MCD < 34 µs was considered normal.

**Table 4:** Electrophysiological comparison of contralateral orbicularis oculi muscle MCD and jitter values in first-time versus recurrent BoNT-A recipients.

Measurement Timepoint	First-Time BoNT Injection (10 Patients)			Recurrent Treatment (29 Patients)			*p*
	Mean ± SD	Med (Min–Max)		Mean ± SD	Med (Min–Max)		
Contralateral Side MCD							
Before BoNT-A	29.2 ± 4.0	29.0	23.0–35.0	34.6 ± 5.5	34.0	22.0–44.0	*0.009 * ^m^
After BoNT-A	31.4 ± 2.8	31.5	28.0–35.0	39.0 ± 4.5	39.0	30.0–48.0	*0.00 * ^m^
Before/After BoNT-A Change	2.2 ± 3.1	2.0	−4.0–7.0	4.8 ± 3.7	4.5	−3.0–11.0	*0.03 * ^m^
Before/After BoNT-A Change *p*	0.049 ^w^			0.000 ^w^			
Contralateral Side Jitter							
Before BoNT-A	2.0 ± 2.6	0.0	0.0–5.0	9.9±5.7	10.0	0.0–24.0	0.000 ^m^
After BoNT-A	5.0 ± 3.3	5.0	0.0–10.0	17.4 ± 6.7	15.0	9.0–30.0	0.000 ^m^
Before/After BoNT-A Change	3.0 ± 2.6	5.0	0.0–5.0	7.3 ± 4.1	5.0	0.0–20.0	0.009 ^m^
Before/After BoNT-A Change *p*	0.014 ^w^			0.000 ^w^			

MCD: mean consecutive difference; ^w^: Wilcoxon signed-rank test; ^m^: Mann–Whitney U test; BoNT-A Botulinum toxin A. Values are presented as mean ± SD and median (min–max).

## Data Availability

The original contributions presented in this study are included in the article. Further inquiries can be directed to the corresponding author.

## Abbreviations

BoNT-A	Botulinum Toxin Type A
CMAP	Compound Muscle Action Potential
EMG	Electromyography
HFS	Hemifacial Spasm
MCD	Mean Consecutive Difference
REZ	Root Exit Zone
SFEMG	Single-Fiber Electromyography
